# Process evaluation of an integrated care pathway in geriatric rehabilitation for people with complex health problems

**DOI:** 10.1186/s12913-016-1974-5

**Published:** 2017-01-13

**Authors:** Irma H. J. Everink, Jolanda C. M. van Haastregt, Jose M. C. Maessen, Jos M. G. A. Schols, Gertrudis I. J. M. Kempen

**Affiliations:** 1 Department of Health Services Research and Care and Public Health Research Institute (CAPHRI), Maastricht University, P.O. Box 616, 6200 MD Maastricht, The Netherlands; 2 Department of Patient & Integrated Care, Maastricht University Medical Centre, P.O.Box 5800, 6202 AZ Maastricht, The Netherlands; 3 Department of Family Medicine and Care and Public Health Research Institute (CAPHRI), Maastricht University, P.O. Box 616, 6200 MD Maastricht, The Netherlands

**Keywords:** Integrated care pathway, Continuity of patient care, Implementation, Process evaluation, Geriatric rehabilitation, Patient transfer, Feasibility studies

## Abstract

**Background:**

An integrated care pathway in geriatric rehabilitation was developed to improve coordination and continuity of care for community-living older adults in the Netherlands, who go through the process of hospital admission, admission to a geriatric rehabilitation facility and discharge back to the home situation. This pathway is a complex intervention and is focused on improving communication, triage and transfers of patients between the hospital, geriatric rehabilitation facility and primary care organisations. A process evaluation was performed to assess the feasibility of this pathway.

**Methods:**

The study design incorporated mixed methods. Feasibility was assessed thru if the pathway was implemented according to plan (fidelity and dose delivered), (b) if patients, informal caregivers and professionals were satisfied with the pathway (dose received) and (c) which barriers and facilitators influenced implementation (context). These components were derived from the theoretical framework of Saunders and colleagues. Data were collected using three structured face-to-face interviews with patients, self-administered questionnaires among informal caregivers, and group interviews with professionals. Furthermore, data were collected from the information transfer system in the hospital, patient files of the geriatric rehabilitation facility and minutes of evaluation meetings.

**Results:**

In total, 113 patients, 37 informal caregivers and 19 healthcare professionals participated in this process evaluation. The pathway was considered largely feasible as two components were fully implemented according to plan and two components were largely implemented according to plan. The timing and quality of medical discharge summaries were not sufficiently implemented according to plan and professionals indicated that the triage instrument needed refinement. Healthcare professionals were satisfied with the implementation of the pathway and they indicated that due to improved collaboration, the quality of care provision improved. Although patients and informal caregivers were also satisfied with the care provision in the pathway, they indicated that the care organisations involved should pay more attention towards providing information about their treatment.

**Conclusions:**

This process evaluation showed that patients, informal caregivers and professionals are fairly satisfied with the care provision in the pathway and professionals reported that collaboration improved. Extra attention should be paid to the components in the pathway that were not implemented according to plan.

**Trial registration:**

ISRCTN90000867 Registered 7 April 2016.

## Background

After hospital discharge, a growing number of older patients are temporarily admitted to an inpatient geriatric rehabilitation facility, where they receive short-term multidisciplinary care to improve physical function, independence and self-care, and to increase or restore participation [1]. A systematic review showed that geriatric rehabilitation indeed has beneficial effects for functional improvement, prevents admissions to nursing homes, and reduces mortality [2]. However, when older adults go through the full trajectory of hospitalisation, admission to a geriatric rehabilitation facility and discharge back to the home situation, they face various challenges [3]. First, because patients transit between care settings, they are confronted with different caregivers, which may threaten continuity of care [4]. Second, transitions in care can lead to problems such as ineffective discharge planning and miscommunication between care providers, patients and informal caregivers [5]. Finally, incomplete discharge information may negatively affect quality of care and patient safety and potentially cause adverse events such as hospital readmission, permanent admission to nursing homes, or even death [6, 7]. To achieve optimal geriatric rehabilitation care, these challenges in continuity and coordination of care need to be addressed [7].

Accordingly, to meet these challenges, an integrated pathway in geriatric rehabilitation was developed in the Maastricht area (southern part of the Netherlands) in the period 2012-2014 for older adults with complex health problems. This pathway is focused on improving communication, triage and transfers of frail older patients between the hospital, geriatric rehabilitation facility and primary care organisations.

Integrated care pathways have traditionally been based on specific conditions in the hospital setting, for example hip fracture [8, 9]. Nowadays, an increasing number of pathways have been developed which focus on the transition of frail older adults and cross the boundaries of care settings. These pathways focus on improving continuity and coordination of care within and between care settings [10–14]. To our knowledge, no integrated care pathway has yet been developed for patients who transfer between more than two settings; therefore this integrated care pathway was developed. This pathway is a complex intervention, targeting multiple interacting components, such as organisational structures, healthcare professionals in various settings, patients and informal caregivers. This makes the implementation a challenging process [15]. To be able to draw conclusions about the feasibility of the pathway, an extensive process evaluation was carried out. The results of this process evaluation are presented in this study. Feasibility was examined by assessing several aspects of the implementation process which were relevant and assessable for the current evaluation, based on the framework laid out by Saunders and colleagues [16]. This is a framework often used for process evaluations of innovations in health care [17–19]. The process factors that were assessed were 1) the extent to which the pathway was implemented as planned (fidelity and dose delivered); 2) the extent to which patients, informal caregivers and healthcare professionals were satisfied with the pathway (dose received - satisfaction), and 3) the influence of external factors (barriers and facilitators) on the implementation of the pathway (context).

## Methods

### Integrated care pathway in geriatric rehabilitation

The pathway was developed using a bottom-up approach. Through literature research and through consultation with experts and care providers, current practice, barriers and incentives for change were systematically analysed. Based on this analysis, three multidisciplinary working groups of patient representatives, informal caregivers and professionals discussed how current care delivery could be optimised. This resulted in concrete proposals for improvement which were critically discussed in all working groups. These proposals were finally combined and included in the integrated care pathway. The development of the pathway is described elsewhere [3]. The pathway consists of 31 specific elements (Appendix 1); five core components can be distinguished. These five core components are:

1. A care pathway coordinator is appointed. The role of the care pathway coordinator is to act as a port of call for professionals involved in the pathway, to improve communication between professionals from different settings, improve continuity and coordination of care and to further streamline the pathway.
2. A triage instrument (Appendix 2) is introduced to be used by discharge nurses in the hospital. This instrument is based on a triage instrument developed by the expert opinion of the Dutch association of elderly care physicians (Verenso) [20]. The instrument instructs discharge nurses to gather information on each patient regarding their functional prognosis, endurability, teachability/trainability and both the patient’s and informal caregiver’s needs and abilities. This information should enable the users of the instrument to decide if geriatric rehabilitation is appropriate for a patient or not. If the discharge nurse has doubts about the appropriateness of geriatric rehabilitation for a patient, an elderly care physician from the geriatric rehabilitation facility should be consulted.
3. Patients and their informal caregivers are always actively involved in the triage decision in the hospital, and in the establishment of their care and treatment plan in the hospital, the geriatric rehabilitation facility and in primary care.
4. All patient discharge summaries (medical and nursing) from the hospital to the geriatric rehabilitation facility and from the geriatric rehabilitation facility to primary care professionals are sent no later than on the day of discharge and should be of high quality.
5. Evaluation meetings between care professionals from the hospital and the geriatric rehabilitation facility are organised at least twice a year, and between the geriatric rehabilitation facility and primary care professionals at least once a year. These meetings should focus on improving the triage process, the timing and quality of discharge summaries and the (quality of the) transfer of patients between the hospital, geriatric rehabilitation facility and primary care.

### Design

This process evaluation used a design incorporating mixed methods, including both qualitative and quantitative data collection methods. Process data were gathered alongside a prospective cohort study on the effects of the pathway. The results of this study of effects will be published elsewhere.

This study design and methods were approved by the Medical Ethics Committee of the University Hospital Maastricht (#11-4-020).

### Setting and participants

This study was conducted in a university hospital, a geriatric rehabilitation facility (which in the Netherlands are usually situated in a nursing home) and primary care organisations in the Maastricht area (southern part of the Netherlands). The study population of this process evaluation consisted of three groups of participants: 1) patients who received care during and after implementation of the pathway; 2) their informal caregivers; and 3) their care professionals in the hospital, the geriatric rehabilitation facility and in primary care. Patients and informal caregivers provided written informed consent and care professionals provided verbal consent for participation in this study.

### Patients

In the Netherlands, four main categories of patients can be distinguished within geriatric rehabilitation: patients with stroke, patients with orthopaedic trauma, elective orthopaedic patients, and a residual group, referred to as geriatric patients with complex health problems with related functional loss and care dependency [21]. The pathway described in the present study was developed for the geriatric patients with complex health problems. This heterogeneous group of patients is often suffering from multi-morbidity, mostly involving cardiac problems, respiratory problems, neurological problems and other internal medicine problems such as gastrointestinal problems. Disease exacerbations are common in this group, leading to hospital readmissions and the need for geriatric rehabilitation.

Patients were eligible for participation if they were admitted to the geriatric rehabilitation facility between April 2013 and August 2014. Furthermore, they had to have been admitted to a hospital prior to rehabilitation in the geriatric rehabilitation facility, aged ≥ 65 years and be community-dwelling. Patients were not eligible for participation if their cognitive ability (assessed by an elderly care physician) was considered insufficient for participation in the study. A trained research assistant recruited patients by visiting all eligible patients in the geriatric rehabilitation facility and asking them if they were willing to participate in the study.

### Informal caregivers

The informal caregiver was defined as the person the patient expects to be their most important informal caregiver after discharge to the home situation (e.g. a family member, friend or neighbour). The informal caregivers were recruited by asking the patients if they had an informal caregiver who could be invited to participate in the study. These informal caregivers were invited for participation by telephone.

### Healthcare professionals

We included care professionals from the various settings who were involved in developing the pathway. These professionals were chosen, based on their involvement in the five key elements of the pathway. These professionals represented the three settings involved: the hospital (discharge nurses), the geriatric rehabilitation facility (elderly care physicians, nurses and physiotherapists) and primary care (specialised nurses working in the practices of general practitioners (GPs) and professionals from home care organisations).

### Data collection

An experienced, trained research assistant conducted three structured face-to-face interviews with patients at admission to the geriatric rehabilitation facility, after three months, and after nine months (April 2013 - June 2015). Face-to-face interviews were chosen over written questionnaires due to the frailty level of the population. Questions were compiled for this study and evaluated the quality of care received in each setting. Informal caregivers received self-administered questionnaires in the period April 2013 - June 2015 to evaluate the care their relatives received in each setting. These questionnaires were also compiled for this study. Furthermore, semi-structured group interviews with healthcare professionals were conducted in the period February 2015 - June 2015. This method was chosen to be able to gather the most relevant information about the implementation process from the perspective of professionals. Two members of the research team (authors IHJE and JCMvH) conducted these group interviews which were focused on the extent to which professionals experienced the pathway as being implemented according to plan, whether or not professionals were satisfied with the pathway elements and if external factors influenced the implementation process. Furthermore, data were retrospectively retrieved from the information transfer system, from patient files of the participating hospital and geriatric rehabilitation facility and from minutes of weekly meetings with the care pathway coordinator and minutes of structural evaluation meetings. Table 1 provides an overview of the data collection methods used.

**Table 1:** Data collection methods

Measurement method							
Concept	Operationalization	IP	QIC	GIP	PF	ITS	M
Implementation according to plan (Fidelity & Dose delivered)	Care pathway coordinator						X
	Triage instrument			X		X	
	Active involvement			X			
	Patient discharge summaries			X	X		
	Structural evaluation meetings			X			X
Satisfaction with the care pathway (Dose received - Satisfaction)	Healthcare professionals			X			
	Patients	X					
	Informal caregivers		X				
Barriers and facilitators influencing implementation (Context)	Barriers or facilitators influencing the role of the care pathway coordinator.						X
	Barriers or facilitators influencing the triage process.			X			X
	Barriers or facilitators influencing involvement of patients and informal caregivers.	X	X	X			
	Barriers or facilitators influencing patient discharge summaries.			X			X
	Barriers or facilitators influencing the organization and content of meetings			X			X

*IP* Interviews Patient, *QIC* Questionnaire Informal Caregiver, *GIP* Group Interview Professionals, *PF* Patient Files, *ITS* Information Transfer System, *M* Minutes of Meetings

### Data analysis

The quantitative data were analysed using the statistical software package SPSS for Windows, version 22. Descriptive statistics were used for frequencies, percentages, means and standard deviations. The continuous demographic variables were analysed using independent t-tests, whereas the ordinal data were analysed using chi square or Fisher’s exact tests. The group interviews with professionals were audio-taped and transcribed by one of the authors (IHJE). The transcripts were systematically read and coded (by author IHJE), which caused major themes to emerge. These themes were linked to the theoretical components of Saunders and colleagues [16] and on the five key pathway elements of the study. Author IHJE checked in the information transfer system if the triage instrument was used for all patients and assessed the timeliness of patient discharge summaries in the patient files. Finally, author IHJE analysed the minutes of the weekly evaluation meetings of authors IHJE and JCMvH with the care pathway coordinator and minutes of the structural evaluation meetings between the hospital, the geriatric rehabilitation facility and primary care organisations to gather additional information on the process components.

## Results

### Sample

**Fig. 1:**
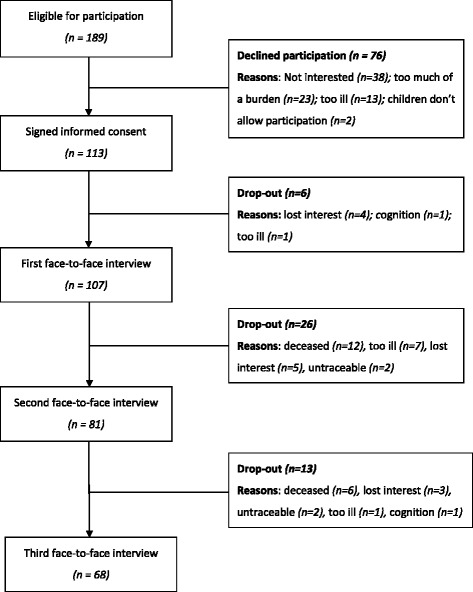
Flowchart patient study population

In total, 189 patients were eligible for participation. Of these 189 patients, 113 patients (60%) were willing to participate in the current study. The mean age of these patients was 81 (SD 6.9) and 32% were male. Furthermore, 69% were living alone before hospital admission and 52% assessed their health as fair or poor (as compared with excellent/very good/good). There were no significant baseline differences between the patients who dropped out of the study (*n* = 45) and patients who completed all measurements (*n* = 68). Figure 1 shows the flowchart of the patient study population.

In relation to the 113 patients who were included in the study, 29 informal caregivers (26%) were willing and able to participate in the study. An additional 8 informal caregivers participated from the group of 76 patients who declined participation, meaning that in total 37 informal caregivers were included in the study. The main reasons for not participating were that (a) the patient indicated not having a caregiver (*n* = 32), (b) the caregiver was not interested in participation (*n* = 24) or (c) the patient did not want to burden the informal caregiver (*n* = 10). The mean age of the informal caregivers was 63 years (SD 15.4), ranging from 19 to 88. The majority of the informal caregivers were female (65%). Of the informal caregivers, 54% were a daughter or son (in law), 22% were spouses, 5% a brother or sister and 19% had another relationship with the patient.

In total, 21 professionals were approached to participate in this process evaluation and 19 participated in the semi-structured group interviews. Six interviews were conducted: with hospital discharge nurses (*n* = 8), with elderly care physicians (*n* = 2), physiotherapists (*n* = 3) and nurses (*n* = 3) at the geriatric rehabilitation facility, with professionals from home care organisations (*n* = 2) and with a specialised nurse working in the GP practice (*n* = 1).

### Implementation according to plan (fidelity and dose delivered)

#### Care pathway coordinator

According to plan, a care pathway coordinator was included in the pathway. The role of the care pathway coordinator was also performed according to plan as the minutes taken during the feedback and evaluation meetings demonstrate that the main focus was on analysing barriers and facilitators in the delivery of care in the pathway, on discussing barriers in communication between professionals and on finding opportunities to stream the pathway further.

#### Triage instrument

The discharge nurses used the triage instrument for 100% of the patients who were referred from the hospital to the geriatric rehabilitation facility. The discharge nurses from the hospital reported in their group interview that they always contacted the elderly care physician when they had doubts about the eligibility of a patient for geriatric rehabilitation. However, the elderly care physicians argued that 10% of the patients admitted to the geriatric rehabilitation facility actually needed another type of follow-up care and therefore, consulting them more often would probably decrease this percentage.

#### Active involvement

According to the discharge nurses, elderly care physicians, nurses, home care providers and the specialised nurse of the GP practice, patients were actively informed and involved in the triage decision, in establishing their care and treatment plan and their rehabilitation goals. The professionals of the home care organisations came to the geriatric rehabilitation facility to do an intake to determine the level and type of formal homecare needed for all patients who requested this. The practice nurse stated that once patients were discharged to the home situation, they verified whether the patients received the help they needed. However, physiotherapists from the geriatric rehabilitation facility stated although they did actively involve patients in establishing rehabilitation goals, not all goals could be addressed during inpatient rehabilitation. When patients were sufficiently rehabilitated to safely return home, their additional rehabilitation goals should be tackled at home with the support of primary care physiotherapists. This is illustrated by the following quote of a physiotherapist: *“Some people indicate that they want to do groceries again. We know this cannot be a rehabilitation goal. (…) Doing groceries can be solved with assistance. If you are that far that you can do groceries yourself, you should have been discharged a long time ago.”*

Furthermore, all professionals indicated that the informal caregiver was not always actively involved; only if the patient agreed to involve the informal caregiver or if this seemed essential considering the patient’s cognitive problems.

#### Patient discharge summaries

The elderly care physicians, nurses, physiotherapists, professionals from home care organisations and the specialised nurse of the GP practice all gave a general judgement about the completeness of information and comprehensiveness (quality) of the patient discharge summaries in the group interviews, based on their own expertise. The elderly care physicians stated that the quality of the medical discharge summaries from the hospital to the geriatric rehabilitation facility varied in quality, with some summaries being quite extensive while others were rather cryptic. A quote of the elderly care physicians illustrating this is the following: *“Some discharge summaries are quite extensive while others are too concise and are of no use. Then you formally received a discharge summary but this has no added value”.*

The quality of the medication lists from the hospital was evaluated as poor by the elderly care physicians, as there were incongruities between medication described in the medical discharge summary and medication described in nursing discharge summary. Both nurses in the geriatric rehabilitation facility and professionals from home care organisations were in general satisfied with the quality of the nursing discharge summary.

Table 2 presents the timing of the discharge summaries. The table shows that respectively 91% of the medical and 65% of the nursing discharge summaries from the hospital to the geriatric rehabilitation facility were sent on time. Of the geriatric rehabilitation facility to primary care, 29% of the medical and 52% of the nursing discharge summaries were sent on time.

**Table 2:** Timing of transfer of medical and nursing discharge summaries

Setting	Hospital – GR^a^				GR – Primary care			
Type of discharge summary	Medical		Nursing		Medical		Nursing	
*n* = 107	N	%	N	%	N	%	N	%
On time (day of discharge)	97	91	70	65	31	29	56	52
Too late (after the day of discharge)	1	1	3	3	67	62	1	1
Not received at all	6	5	5	5	4	4	5	5
Unknown (no date on the document)	3	3	29	27	-	-	38	35
Not applicable^b^	-		-		5	5	7	7

^a^ *GR* Geriatric rehabilitation

^b^Not applicable means that the patient is either deceased, readmitted in the hospital or does not need home care

#### Structural evaluation meetings

The frequency of structural evaluation meetings was according to plan in 100% of the meetings and the minutes of these meetings reveal that, as intended, they were focused on providing feedback concerning the triage process and managing patient expectations, solving obstacles in the timing and quality of discharge summaries and on improving the care process. Furthermore, the people who were supposed to attend the structural evaluation meetings were indeed present: at least one representative of the organisations involved in the pathway attended each meeting.

### Satisfaction with the pathway (dose received - satisfaction)

#### Healthcare professionals

The role of the care pathway coordinator was received as satisfactory by representatives of the geriatric rehabilitation facility and home care organisations; they stated in their interviews (where the care pathway coordinator was not present) that the care pathway coordinator succeeded in bringing the professionals from various organisations together and in initiating meetings to improve collaboration and continuity of care. Still, hospital discharge nurses felt that the collaboration and intensity of contact between the hospital and the geriatric rehabilitation facility did not change after implementation of the pathway.

Healthcare professionals from the hospital and the geriatric rehabilitation facility expressed their level of satisfaction with the triage instrument in the interviews. In general, professionals considered the use of the triage instrument as an improvement as they stated that after implementation of the triage instrument, more patients were correctly referred to the geriatric rehabilitation facility. However, professionals in both settings also stated that the triage instrument did not sufficiently discriminate for patients with cognitive problems. Although cognition is assessed in the triage instrument using the component ‘teachability/trainability’, professionals argued that there are no clear criteria regarding the extent to which someone needed to be teachable or trainable. This is demonstrated by the following quote of a discharge nurse: *“There are always dubious cases where the triage instrument is not conclusive. (…) Hospital physicians, discharge nurses and elderly care physicians all interpret it differently.”*

Regarding satisfaction with the patient discharge summaries, the elderly care physicians argued that the medical discharge summaries from the hospital were often incomplete. The elderly care physicians also expressed dissatisfaction with the timing of their own medical summaries at the point of discharge towards primary care. Lack of time was their reason for often sending their own discharge summaries too late. The nurses from the geriatric rehabilitation facility were fairly satisfied with the quality of the nursing discharge summaries and the home care organisations were also genuinely satisfied with the combination of both oral and written discharge summaries received from the geriatric rehabilitation facility. This is illustrated by the following quote from a professional of a home care organisation: *“The information in the nursing discharge summaries we receive is always complete. (…) We never hear colleagues complain about missing information anymore, which used to be different in the past”.*

The specialised nurse of the GP practice stated that although the quality and timing of discharge summaries has improved in comparison with some years ago, there were still patients who were discharged to their home without a medical discharge summary.

In their interviews, the discharge nurses and elderly care physicians expressed that they were satisfied with the content and frequency of the structural evaluation meetings between hospital and geriatric rehabilitation facility and that the meetings were valuable, as they were focused on improving the triage process and the transfer of patients between the settings. This improved mutual understanding and enabled the participants to provide constructive feedback. The professionals from the home care organisations also experienced the evaluation meetings with professionals from the geriatric rehabilitation facility as useful, not only to provide feedback on the current state of affairs but also to discuss future developments in health care.

#### Patients and informal caregivers

Table 3 shows to what extent patients and informal caregivers were satisfied with the care received in the hospital, in the geriatric rehabilitation facility and in primary care, and whether or not they felt that they have benefited from it. As shown in Table 3, more than 80% of the patients assessed the treatment received in all settings as excellent or good. Among informal caregivers, this percentage was more than 57%. Although in general patients were more positive than their informal caregivers, both patients and informal caregivers recognised that the treatment received in all three settings had a beneficial impact on the patient’s health status.

**Table 3:** Satisfaction among patients and informal caregivers with the rehabilitation trajectory

	Setting:	Hospital		Geriatric rehabilitation		Primary care	
	Respondents:	Patients	Informal caregivers	Patients	Informal caregivers	Patients	Informal caregivers
		*n* = 101	*n* = 28	*n* = 74	*n* = 25	*n* = 60	*n* = 15
Satisfaction with treatment received	Excellent/good	72%	57%	84%	64%	80%	62%
	Sufficient	14%	36%	4%	24%	18%	38%
	Fair/poor	14%	7%	12%	12%	2%	0
		*n* = 90	*n* = 73	*n* = 60	*n* = 27	*n* = 25	*n* = 11
Perceived benefit from treatment received	Excellent/good	89%	66%	85%	72%	85%	100%
	Sufficient	4%	30%	7%	12%	7%	0
	Fair/poor	7%	4%	8%	16%	8%	0

Patients and informal caregivers were also asked whether or not they felt that their personal needs and wishes were sufficiently taken into account in the hospital, in the geriatric rehabilitation facility and in primary care (Table 4). A substantial percentage of informal caregivers were not satisfied with the extent to which their personal needs and wishes were taken into account in the hospital (43%) and with the information provided regarding care and treatment in the hospital (36%) and primary care (40%). More specifically, they were not satisfied due to a lack of communication from professionals towards the patient and the family, and because there was insufficient personal attention paid towards the patient.

**Table 4:** Patients and informal caregivers’ experience with involvement in decision-making

	Setting	Hospital		Geriatric rehabilitation		Primary care	
	Respondents	Patients	Informal caregivers	Patients	Informal caregivers	Patients	Informal caregivers
		*n* = 85	*n* = 28	*n* = 72	*n* = 25	*n* = 58	*n* = 15
Personal needs and wishes taken into account	Excellent/good	71%	39%	87%	44%	81%	33%
	Sufficient	13%	18%	6%	28%	16%	40%
	Fair/poor	16%	43%	7%	28%	3%	27%
		*n* = 98	*n* = 28	*n* = 75	*n* = 25	*n* = 56	*n* = 15
Information provided about care and treatment	Excellent/good	65%	28%	76%	40%	80%	40%
	Sufficient	9%	36%	8%	40%	11%	20%
	Fair/poor	26%	36%	16%	20%	9%	40%
				*n* = 86	*n* = 26		
Involvement in establishing rehabilitation goals	Excellent/good	na	na	77%	31%	na	na
	Sufficient	na	na	8%	42%	na	na
	Fair/poor	na	na	15%	27%	na	na

### Barriers and facilitators influencing implementation (Context)

The external factors facilitating the implementation of the pathway can be categorised into barriers and facilitators and were related to the professional, organisational and political contexts. A facilitator related to the professional context was higher management’s support with the changes required, as minutes revealed that they had committed themselves to the changes proposed by the care pathway. Furthermore, minutes of the weekly meetings with the care pathway coordinator revealed that the independence of the care pathway coordinator was appreciated in her role as a facilitator. Because she was not employed at one of the organisations involved, she could be highly critical about the processes in all organisations and could freely propose changes. Professionals of the home care organisations reported that meetings between themselves and nurses of the geriatric rehabilitation facility were an organisational facilitator. During these meetings, the professionals worked together to improve the content of the nursing discharge summary, resulting in a new discharge summary of higher quality. Finally, the legislative changes in 2013, when the heretofore fully nationally insured geriatric rehabilitation came under a new health insurance modality, were considered to be a facilitator in implementing the pathway related to the political context. Professionals from the hospital and the geriatric rehabilitation facility stated that the changes enforced stricter admission rules for geriatric rehabilitation and therefore, the need to apply the new triage rules was more pressing.

Barriers of the implementation process were related to the innovation (the pathway) and to the organisational context. A barrier related to the innovation (the pathway) was that the triage instrument was not 100% conclusive, resulting in disagreements between professionals from the geriatric rehabilitation facility and the hospital about the referral of specific patients.

The spread of patients all over the hospital and thus the high number of professionals involved in the pathway was regarded as an important organisational barrier to successful implementation, as it was impossible to actively involve all professionals. Finally, the spread of professionals over different locations (hospital, geriatric rehabilitation facility and primary care organisations) made it difficult to organise structural evaluation meetings where all representatives could be present.

## Discussion

The integrated care pathway consists of five core components: 1) the appointment of a care pathway coordinator; 2) the use of a triage instrument by discharge nurses in the hospital; 3) the active involvement of patients and their informal caregivers; 4) the timeliness and high quality of patient discharge summaries and 5) the organisation of structural evaluation meetings between the hospital, the geriatric rehabilitation facility and primary care.

The process evaluation of this pathway revealed that the pathway was largely feasible. When answering the first research question, to what extent has the pathway been implemented according to plan (fidelity and dose delivered), we can conclude that the appointment of a care pathway coordinator and the organisation of structural evaluation meetings between care professionals were fully implemented according to plan. The use of a triage instrument by the discharge nurses under the responsibility of an elderly care physician and the active involvement of patients and informal caregivers were partly implemented according to plan. Finally, the timeliness and quality of the medical discharge summaries has not sufficiently been implemented according to plan, as the quality of medical discharge summaries was rather variable and a large percentage of medical discharge summaries from the geriatric rehabilitation facility to primary care were sent too late.

When it comes to answering the second research question, to what extent were patients, informal caregivers and healthcare professionals satisfied with the pathway (dose received – satisfaction), we can conclude that patients were fairly satisfied with their rehabilitation trajectory, as more than 70% of the patients appraised the treatment received in the hospital, the geriatric rehabilitation facility and primary care as excellent or good. Furthermore, more than 84% of all patients mentioned that they benefited (very) much from the treatment received in the three previously mentioned settings. Still, as mentioned before, more consideration should be given to providing information about the treatment. Healthcare professionals were satisfied with the pathway components and indicated that due to the pathway’s implementation, both the contact and communication between professionals improved, resulting in improved continuity of care. Finally, in the third research question, the influence of professional, social, organisational and political factors was assessed and it appeared that mainly the political context was a facilitator in implementing the pathway.

This pathway is a unique programme for older adults and the healthcare professionals who care for them. Where most integrated care programmes only focus on the hospital and/or primary care [14, 22, 23], this pathway includes geriatric rehabilitation as well. Experiences with such a pathway have not previously been described. Furthermore, very few studies of integrated care interventions across the hospital – primary care continuum performed a detailed process evaluation [24]. A study by Rosstad and colleagues showed that the pathways improved collaboration between professionals but that implementation was demanding and required a lot of work [24]. A study focusing on providers’ perceptions of delivering integrated care found that professionals’ bottom-up involvement during implementation is key to success [25]. Although the interventions implemented in both studies were different from our integrated care pathway, these findings are in line with the results of our evaluation.

We used the conceptual framework of Saunders and colleagues to assess different aspects of the pathway’s feasibility and data was collected from multiple data sources which enabled comprehensive evaluation of the pathway. By collecting data from patients, informal caregivers, professionals from different settings and also from databases, the overall view on feasibility is fairly complete and the possibility of bias is reduced.

### Limitations

Some limitations of this study should also be mentioned. First, not all five core components of the pathway could be assessed objectively. It was difficult to measure whether or not patients and informal caregivers were actively involved because ‘active involvement’ is difficult to define and to assess. The same holds for the word ‘doubt’ when assessing if elderly care physicians were always consulted when there was doubt about eligibility for geriatric rehabilitation during triage.

Second, the timeliness of discharge summaries could not be assessed in all cases because the date when the discharge summary was received could not always be verified. Neither was it possible to assess the timeliness of medication lists and the physiotherapeutic discharge summaries because the medication lists were not sent directly to the geriatric rehabilitation facility, but first to the pharmacy, and also because it was not clear how many patients had visited a physiotherapist in the hospital and how many patients had not. Third, the discharge nurses, the elderly care physicians, the professionals from home care organisations and the specialised nurse of the GP practice had already been actively involved in developing the pathway. Therefore, their answers might have been different from professionals who provide care along the pathway but who had not been involved in this development. Fourth, only a small number of informal caregivers were interested in participating in the study (*n* = 37), which could have led to non-response bias if these participants are not representative of the whole group of informal caregivers. Finally, all participants – patients, informal caregivers and professionals - might have given socially desirable answers. We tried to avoid this by stressing among patients that their answers would be treated confidentially and that study participation would not affect their (right to) healthcare services. Professionals were assured that the interviews were conducted in order to assess the effects of the pathway, not to criticise their competencies.

## Conclusions

Based on the results of this process evaluation it seems that the pathway as we have designed it is largely feasible. Professionals are fairly satisfied with the content of the pathway and with the extent to which the pathway is used in regular care. However, special attention should be paid to four aspects. First, we recommend critical revision of the cognition component (teachability/trainability) in the triage instrument and also developing clearer admission criteria for patients with cognitive problems. This should make the triage process more transparent. Second, we recommend improving the provision of information in the hospital, the geriatric rehabilitation facility and in primary care to both informal caregivers and to patients about their treatment. Third, the quality and timing of medical discharge summaries from the hospital to the geriatric rehabilitation facility and from the geriatric rehabilitation facility to primary care should be improved. We recommend initiating this by organising one or more meetings between physicians from the hospital, the geriatric rehabilitation facility and primary care. During these meetings, they should discuss which information is needed in the discharge summaries and what timing is necessary to safely provide follow-up care. The possibilities of using technology when transferring discharge summaries could also be explored. Finally, as professionals in the pathway work in different areas, digital resources (such as videoconferencing) could also facilitate the organisation of structural evaluation meetings and this option should be explored.

## Figures and Tables

**Fig. 2:**
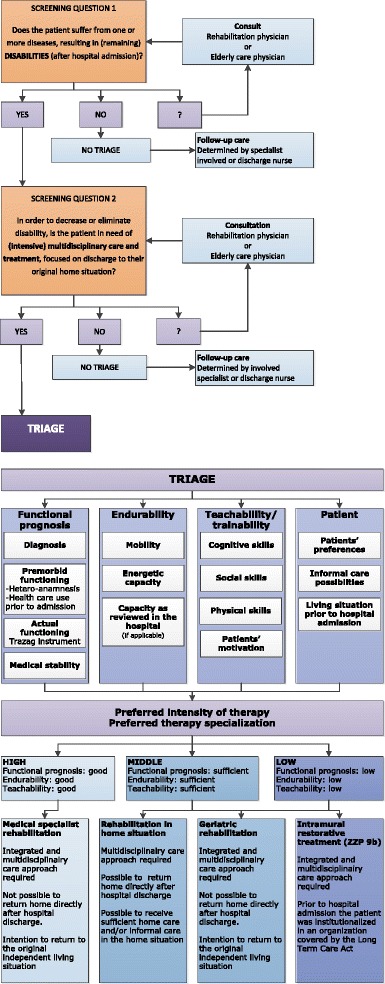
Screening questions and triage instrument

## Appendix 1

**Table 5:** Integrated care pathway for geriatric rehabilitation

Setting	No.	Care pathway element
Hospital	1	If the main treatment provider believes that the patient is eligible for geriatric rehabilitation, the discharge nurses of the hospital will be consulted. Preferably, this consultation takes place well in advance of discharge.
	2	Dismissal from the hospital is preceded by a triage by a discharge nurse. Information about the patient's functional prognosis, endurability, teachability and trainability and the patient’s and informal caregiver’s needs and abilities needs to be gathered to make this triage decision.
	3	The triage is always performed under the responsibility of an elderly care physician from the geriatric rehabilitation facility. If the discharge nurse has doubts about eligibility of the patient for geriatric rehabilitation, the elderly care physician should be consulted.
	4	Information about functional prognosis, endurability, teachability and trainability and needs and abilities of the patient should be gathered by consulting professionals in the hospital who have been involved in the patient’s care.
	5	The patient should always be asked about their needs and abilities and this should explicitly be taken into account when making the triage decision.
	6	The informal caregiver should (if applicable) be asked about their ability to provide informal care and this should explicitly be taken into account when making the triage decision.
